# Correlative vibrational spectroscopy and 2D X-ray diffraction to probe the mineralization of bone in phosphate-deficient mice

**DOI:** 10.1107/S1600576719009361

**Published:** 2019-08-23

**Authors:** Helen E. King, Steven M. Tommasini, Alejandro B. Rodriguez-Navarro, Brandon Q. Mercado, H. Catherine W. Skinner

**Affiliations:** a Department of Earth Sciences, Utrecht University, Princetonlaan 8a, Utrecht 3584 CB, The Netherlands; b Department of Geology and Geophysics, Yale University, 210 Whitney Avenue, New Haven, Connecticut CT-06511, USA; c Department of Orthopaedics and Rehabilitation, Yale University School of Medicine, 330 Cedar Street, New Haven, Connecticut CT-06510, USA; d Departmento de Mineralogia y Petrologia, Universidad de Granada, Granada 18002, Spain; e Department of Chemistry, Yale University, 225 Prospect Street, New Haven, Connecticut CT-06511, USA

**Keywords:** 2D-XRD, vibrational spectroscopy, bone remodelling, hypophosphatemia

## Abstract

Bone crystallite chemistry and structure change during bone maturation. However, these properties of bone can also be affected by limited uptake of the chemical constituents of the mineral by the animal. This makes probing the effect of bone-mineralization-related diseases a complicated task. Here it is shown that the combination of vibrational spectroscopy with two-dimensional X-ray diffraction can provide unparalleled information on the changes in bone chemistry and structure associated with different bone pathologies (phosphate deficiency) and/or health conditions (pregnancy, lactation). Using a synergistic analytical approach, it was possible to trace the effect that changes in the remodelling regime have on the bone mineral chemistry and structure in normal and mineral-deficient (hypophosphatemic) mice. The results indicate that hypophosphatemic mice have increased bone remodelling, increased carbonate content and decreased crystallinity of the bone mineral, as well as increased misalignment of crystallites within the bone tissue. Pregnant and lactating mice that are normal and hypophosphatemic showed changes in the chemistry and misalignment of the apatite crystals that can be related to changes in remodelling rates associated with different calcium demand during pregnancy and lactation.

## Abstract 2

2D X-ray diffraction and vibrational spectroscopy are used to follow changes in bone remodelling due to different health conditions (pregnancy, lactation) and bone pathologies (phosphate deficiency) in mice.

## 1. Introduction   

Bone is a complex inorganic–organic hybrid material, comprising nano-sized carbonate apatite crystals mineralizing a collagen matrix. It serves multiple functions within the body, including structural support for our locomotive system and as an ion reservoir for homeostasis (Glimcher, 2006 ▸). However, bone metabolic diseases and/or malnutrition can disrupt the ability of the organism to mineralize the collagen matrix, causing skeletal malformations such as rickets or osteoporosis. Severe genetic diseases such as X-linked hypophosphatemia (XLH) are characterized by increased activity of the enzyme alkaline phosphatase and increased renal phosphate wasting, resulting in poor bone-matrix mineralization (Carpenter *et al.*, 2011 ▸). Lower Ca, and specifically phosphate, absorption in mice with a phenotype of XLH (HYP mice) has been shown to correlate with higher carbonate content in the bone (Macica *et al.*, 2016 ▸) and dentine (Amenta *et al.*, 2018 ▸) minerals. These changes become more evident in bone during conditions of high calcium demand, such as pregnancy or lactation, during which bone remodelling increases to meet increasing calcium demand (Macica *et al.*, 2016 ▸).

Increasing the carbonate content of bone apatite crystals is expected to have consequences for their physical properties and functionality. For example, carbonate incorporation into synthetic hy­droxy­apatites has been shown to decrease the crystallinity (McElderry *et al.*, 2013 ▸) and thus stability of the mineral phase (Rollin-Martinet *et al.*, 2013 ▸). In addition, higher carbonate contents have been shown to increase resorption by osteoclast cells (Nakamura *et al.*, 2016 ▸). Therefore, probing the consequences of changes in bone mineral chemistry is an important area of research to understand the impact of altered mineralization on bone functionality. However, bone mineralization is a complex process that has been shown to occur in two stages, where fast mineralization of up to 75% of the final mineral content in bone occurs initially (primary mineralization), followed by a slower secondary mineralization step (Busa *et al.*, 2005 ▸; Bala *et al.*, 2010 ▸; Boivin & Meunier, 2002 ▸). Secondary mineralization creates highly oriented crystals aligned with the collagen fibres. Therefore, newly deposited bone mineral is expected to be poorly organized with nearly random crystal orientations compared with more mature bone tissue with a higher degree of crystal alignment (Ishimoto *et al.*, 2013 ▸). Similarly, the bone crystals themselves undergo chemical and structural modification during maturation (Glimcher, 1998 ▸). In normal bone, mineral maturation is associated with an increase in crystallinity, but also with a concomitant increase in carbonate substitution (Combes *et al.*, 2016 ▸). Thus, deconvoluting the effects of maturation and inhibited mineralization is not trivial. Identifying mature and immature bone and linking this to changes in chemistry and structure on the mineral and tissue scale requires the physical separation of mineral fractions (Rey *et al.*, 2009 ▸) or a synergistic approach of complementary techniques that probe these fractions independently.

X-ray diffraction (XRD) and vibrational spectroscopy (Raman and IR) are routinely used in the analysis of bone to study its crystallinity (Bonar *et al.*, 1983 ▸; Kuhn *et al.*, 2008 ▸) and chemical changes (Penel *et al.*, 1998 ▸; Awonusi *et al.*, 2007 ▸; Cazalbou *et al.*, 2004 ▸; Rey *et al.*, 1989 ▸; Macica *et al.*, 2016 ▸), respectively. This includes studying changes in chemistry or crystallinity associated with bone mineralization disorders; see Boskey (2002 ▸) for a short review. However, these techniques also produce complimentary information about structural ordering within the crystallites (Querido *et al.*, 2018 ▸; Londoño-Restrepo *et al.*, 2019 ▸), as vibrational spectroscopy is sensitive to short-range order (Wopenka & Pasteris, 2005 ▸), whereas XRD can be used to examine changes in long-range order (Bazin *et al.*, 2009 ▸). Whilst vibrational spectroscopy can be conducted on cross-sectioned bone samples, XRD analysis is typically conducted in powder form, resulting in a loss of spatial information. In addition, neither vibrational spectroscopy nor powder XRD provides information about the structural properties of the bone tissue itself, which is critical for evaluating the maturation stage of the bone site investigated. In contrast, two-dimensional XRD (2D-XRD) can be conducted with no additional sample preparation and on the same cross sections used for vibrational spectroscopy. This technique also provides information on the crystallinity of different bone mineral fractions independently and on the degree of crystal alignment within the bone tissue (Dominguez-Gasca *et al.*, 2019 ▸; Rodriguez-Navarro *et al.*, 2018 ▸). Here we test the use of 2D-XRD for studies of bone mineralization disorders and demonstrate that the combination of vibrational spectroscopic techniques and 2D-XRD analysis provides unparalleled insights into bone properties that are relevant for these disorders.

## 2. Experimental details   

### 2.1. Sample preparation   

**Figure 1:**
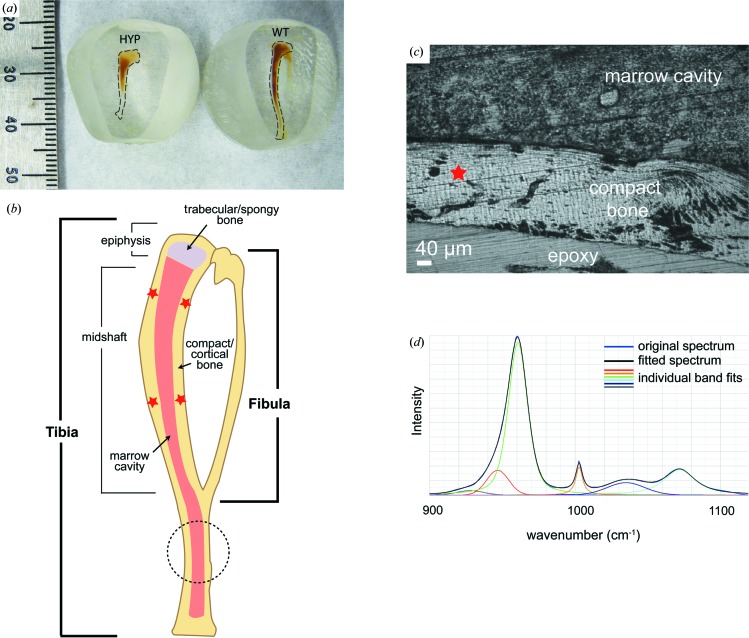
(*a*) The sampling areas for Raman and XRD analysis, showing a comparison of wild-type (WT) and hypophosphatemic (HYP) mouse tibia and fibula longitudinal cross sections embedded in (poly)methyl methacrylate blocks. The tibia are highlighted by dashed lines. (*b*) A schematic diagram of the tibia and fibula, showing the approximate positions of the Raman maps (stars) and 2D-XRD analysis (dashed circle). (*c*) A light microscopy image of a typical Raman map site (star) from a midshaft site on an HYP mouse bone. (*d*) A typical fit of a Raman spectrum taken from a WT mouse bone sample.

Hypophosphatemic (HYP) and normal wild-type (WT) mice were used in this study (Table 1 ▸ and Fig. 1 ▸); see Macica *et al.* (2016 ▸) for details of the animal rearing and bone sampling. The experimental conditions, animal age and reproductive age were selected to provide insights into the effect of metabolic condition on bone mineral quality. Mice are breast-fed for the first three weeks of life, so at two weeks of age are still dependent on maternal milk for all minerals in their diet. During this stage HYP mice do not display any metabolic or skeletal phenotypes associated with hypophosphatemia, as all mineral demand is met by breast milk. Similarly, all mineral demand in WT mice is also met by the consumption of breast milk during this phase. At five months of age, the mice are skeletally mature and the HYP mice display the effects of hypophosphatemia. Thus, five-month-old mice were selected so that the effects of increased mineral demand, through pregnancy and lactation, on mineral quality could be examined. Virgin HYP mice sacrificed at five months of age were used as a baseline for mineral quality. Mineralization of foetuses is highest during late pregnancy (E10–E18.5), therefore by sacrificing the mice at the end of pregnancy (day of giving birth, E18.5) maternal mineral demand is captured in the pregnant mouse specimens. Similarly, the lactating specimens were shown to display the bone remodelling and hormonal balance effects of lactation at 21 days after giving birth.

The mice were reared in accordance with the Guide for the Care and Use of Laboratory Animals recommendations, and the procedural protocol was approved by Yale’s Institutional Animal Care and Use Committee.

A longitudinal cross section of the right tibia and fibula bones embedded in a (poly)methyl methacrylate (PMMA) matrix was used for Raman spectroscopic and 2D-XRD analyses. For Fourier transform infra-red (FT–IR) analysis, left femoral diaphyses were embedded and microtomed to produce longitudinal thin sections of 4 µm thickness.

### 2.2. X-ray diffraction analysis   

**Figure 2:**
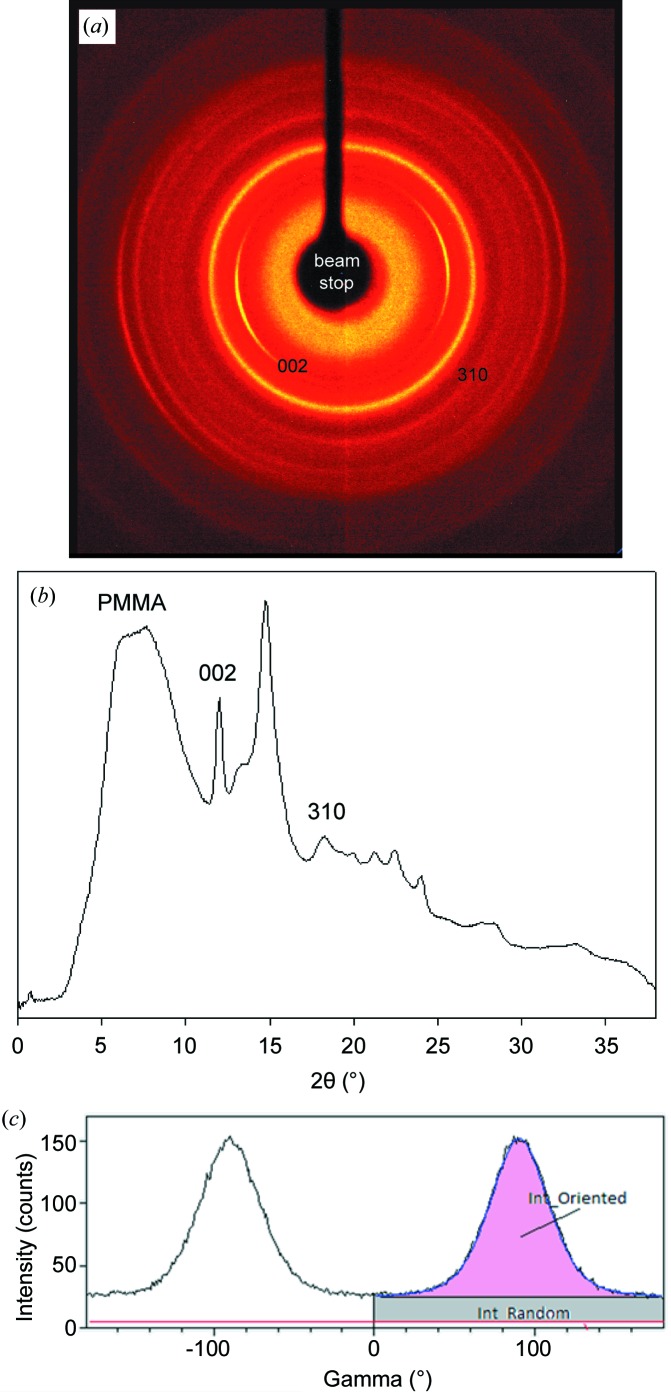
(*a*) A representative 2D-XRD pattern generated from an orientated pregnant WT mouse bone sample. Arcs displayed in the 002 ring show that the crystal *c* axes are aligned with the long axis of the bone. (*b*) A 2θ scan generated from an angular sector in the 2D-XRD pattern containing the intense 002 arc. The large peak beginning at 5° is related to the (poly)methyl methacrylate (PMMA) block that the bone was embedded in. No background removal has been conducted on either of the patterns. (*c*) A 002 γ scan showing the contribution of the oriented and non-oriented fractions of bone mineral.

Whole bone samples were analysed in transmission mode with an X-ray single-crystal diffractometer (D8 VENTURE, Bruker) equipped with a PHOTON 100 area detector and Mo microsource (50 kV and 1 mA, wavelength 0.71 Å; 0.1 mm collimator). Apatite crystals in cortical bone generally show a preferential orientation with their *c* axes aligned parallel to the elongation axis of long bones (Weiner & Wagner, 1998 ▸), visible as two arcs in the Debye–Scherrer ring [Fig. 2 ▸(*a*)]. A quantitative estimate of the degree of alignment of the crystals’ *c* axes was determined from the angular breadth of bands displayed in the intensity profile along the Debye–Scherrer ring (γ scan) for the 002 reflection of apatite (Wenk & Heidelbach, 1999 ▸). A narrower band indicates a higher degree of crystal alignment. The intensity below the two bands corresponds to randomly oriented crystals contributing as a continuous ring to the 2D-XRD patterns [Fig. 2 ▸(*c*)]. The fraction of oriented bone crystals (FOBC) can be estimated as *I*_oriented_/(*I*_oriented_ + *I*_random_), where *I*_oriented_ is the 002 intensity of the bands and *I*_random_ is the intensity of the continuous ring (Dominguez-Gasca *et al.*, 2019 ▸).

Data reduction including centring and 2θ and γ scan generation was performed using the *XRD2DScan* software (Version 4.1.1; Rodriguez-Navarro, 2006 ▸). The peaks in the 2θ scans and bands in the γ scans were fitted in *Fityk* (Wojdyr, 2010 ▸) using a Gaussian function. Additionally, crystallinity was determined by measuring the FWHM of the 002 and 310 peaks displayed in the 2θ scans [Fig. 2 ▸(*b*)] using the Scherrer equation to calculate the crystallite domain size for each reflection (*t_hkl_*) (Langford & Wilson, 1978 ▸):
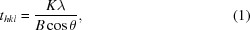
where *B* is the FWHM of the peak in radians. *K* was estimated to be 2.0 (Ziv & Weiner, 1994 ▸).

Standard carbonated apatite powder samples were examined under a microscope and mounted with oil on a MiTeGen mount, then transferred onto an AFC11 goniometer. Room-temperature diffraction data (Fig. S1 in the supporting information) were collected with a Rigaku MicroMax-007HF source (Cu *K*α; λ = 1.54178 Å) coupled to a Saturn994+ CCD detector. Line profiles of the 2D diffraction data were integrated and processed with the Rigaku *2DP* software package.

### 2.3. Vibrational spectroscopy analysis   

A Horiba HR800 Raman microspectrometer equipped with a 532 nm laser and 100× objective lens was used to examine spatially resolved areas of cortical bone (Fig. 1 ▸). To reduce the spectral background and prevent sample burning, the laser power was lowered by 1000× using an optical density filter prior to interaction with the sample. The laser beam spot size with the 100× objective lens is ∼1 µm. For spectrum acquisition, a confocal hole size of 300 µm was used with an 1800 grooves mm^−1^ grating and 50 µm slit width.

Four map grids of 4 × 4 spot analyses at a step size of 3 µm were obtained for each sample. Each map area was chosen to lie between osteocytes and to avoid the unmineralized areas reported within HYP mouse cortical bone (Macica *et al.*, 2016 ▸). Each spectrum was collected for 30 s and integrated ten times to obtain adequate signal-to-noise ratios for data analysis. An estimate of the contribution from the PMMA embedding material was calculated from the relative band heights in pure PMMA surrounding the bones. The synthetic standards of *B*-site-substituted carbonated hy­droxy­apatite used for carbon­ate calibration were Biorad hy­droxy­apatite and previously analysed samples (Yoder *et al.*, 2012 ▸). Each of the seven standard samples was in powder form and three spectra were collected within each sample.

Bands from the bone and synthetic samples were fitted on the basis of the band assignments from previous studies (Awonusi *et al.*, 2007 ▸; Pasteris & Ding, 2009 ▸). Prior to calculating the carbonate content from the Raman spectra the PMMA contribution to the spectra was subtracted. First the background in both the PMMA and bone spectra was removed using a linear function in *Labspec 5* (Horiba Scientific). The contribution from the PMMA was then estimated by normalizing the band intensity at 813 cm^−1^ in the bone spectra, the most intense PMMA band in the region of interest (Fig. S2), with that of the pure PMMA spectrum. Finally, the PMMA spectrum was subtracted from the bone spectra using the spectral correction tool also available in *LabSpec 5*. In the analysed samples, some spectra showed evidence for excessive PMMA contribution, which swamped the bone-related spectral bands. These spectra were not used for data analysis.

All of the mouse sample sites analysed using Raman spectroscopy produced spectra with the same band locations within the spectral resolution of the Raman instrument, with slight variations in relative intensities. As documented in other studies (McElderry *et al.*, 2013 ▸; Penel *et al.*, 1998 ▸), the phosphate ν_1_ stretching band was asymmetrical in shape with a shoulder at lower wavenumbers. Therefore, the best fit was found when a band with a centre at 960 cm^−1^ and a second smaller shoulder band close to 950 cm^−1^ were used in the fitting procedure. The phosphate ν_3_ region between 1010 and 1050 cm^−1^ was fitted using two bands, one close to 1025 cm^−1^ and a second close to 1045 cm^−1^ [Fig. 1 ▸(*d*)]. The smaller band at 1010 cm^−1^ reported in other studies and observed in the synthetic samples was often swamped by the larger 1003 cm^−1^ band, resulting in minimal to no contribution detectable for this band in the mouse spectra during our band-fitting procedure. In previous studies the band at 1003 cm^−1^ has been attributed to HPO_4_ sites in the bioapatite crystals that can charge compensate for the inclusion of the carbonate group (Penel *et al.*, 1998 ▸). However, Raman spectra taken for virgin mice that were terminated at two weeks show that an increased intensity in the 1003 cm^−1^ band correlates with the increased intensity in bands throughout the spectral region of interest that are associated with collagen molecules (Fig. 1 ▸). This is in keeping with the expected lower mineralization of the still-developing mouse at two weeks and the attribution of this band to phenyl­alanine groups (Frushour & Koenig, 1975 ▸) of collagen by Pasteris & Ding (2009 ▸). Therefore, we examine the contribution of the 1003 cm^−1^ band as a ratio with the phosphate to examine changes in collagen and HPO_4_ content.

The carbonate contribution produces bands at 1100 and 1070 cm^−1^, depending on whether the carbonate is substituted for an OH (*A*-type) or phosphate (*B*-type) within the hy­droxy­apatite crystal structure, respectively (Penel *et al.*, 1998 ▸). This previous work demonstrated that bone contains both *A*- and *B*-type substitutions. The carbonate contribution was calculated as described in detail by Awonusi *et al.* (2007 ▸) with the inclusion of the *A*-type carbonate contributions using the ratio between the areas of the carbonate and phosphate ν_1_ stretching bands at 1070 (*B*-type) and 1100 cm^−1^ (*A*-type) and 960 (*B*-type) and 950 cm^−1^ (*A*-type), respectively.

The presence of collagen bands can have important consequences for further analysis of carbonate content and crystallinity, where underlying collagen bands may alter the shape and intensity of the mineral-related bands. However, the two-week-old WT mouse shows the largest contribution of the 1003 cm^−1^ band, as can be observed from the spectrum (Fig. S3), but one of the lowest calculated overall carbonate contents. Therefore, the effect of underlying collagen spectra on the crystallinity and carbonate calculation is expected to be minimal.

Errors corresponding to fitting of the spectra were estimated by independently assigning band positions and fitting the spectral region of interest six times. This error was much smaller than the deviation associated with measuring different locations in the bone. Thus, the error associated with calculation of mean values was taken from the standard deviations (s.d.s) across the mapped regions. The synthetic standards’ spectra were fitted using six bands at 948, 960, 1010, 1025, 1045 and 1070 cm^−1^, as described by Awonusi *et al.* (2007 ▸). No evidence for *A*-site substitution of carbonate was found in the synthetic material. Errors in the carbonate content of the mouse specimens were calculated using the s.d.s of the average measurements and the standard error of the slope and intercept generated from linear regression of the calibration plot [Fig. S1(*c*)].

For FT–IR analysis, a synchrotron-based FT–IR microspectroscope with FT–IR imaging was used at Brookhaven National Laboratory National Synchrotron Light Source on beamline U10B. A spectrophotometer, with a Hyperion IR microscope (Bruker, Bilerica, USA) with a mercury cadmium telluride detector, was used in the frequency range 4000–650 cm^−1^ via spectral mapping. Spectra were collected at 128 scans per point in transmission mode. Background spectra were collected through an empty specimen holder. Four 64 × 64 µm regions of interest were analysed on the bone samples. Data were collected and processed using the *Opus* software (Bruker, Bilerica, USA). See Acerbo *et al.* (2012 ▸) and references therein for further details of the experimental setup.

Prior to band fitting, the PMMA-related background was removed using the *Opus* software. To quantify the contribution of the PMMA, a spectrum of the PMMA embedding material alone was taken. PMMA has major absorption features that overlap with the protein, carbonate and mineral phosphate bands found in bone, and is most easily recognized by a unique and intense carbonyl ester absorption band centred at 1745 cm^−1^ (Acerbo *et al.*, 2012 ▸). As described by Acerbo *et al.* (2012 ▸), the mineral-to-collagen ratio was calculated using the intensity of IR bands between 1600 and 1700 cm^−1^ (amide I band) related to the collagen, and the intensity of the phosphate symmetrical stretching bands between 900 and 1200 cm^−1^. The overall carbonate-to-phosphate ratio was calculated using the carbonate (1414–1424 cm^−1^) and phosphate (900–1200 cm^−1^) bands.

### 2.4. Statistical analysis   

Basic descriptive statistics were used to characterize the bone properties. Pearson’s correlation analysis and linear regression models were used to study the relationships between the different properties of bone. One-way and two-way analysis of variance (ANOVA) tests were used to determine differences among groups for all determined bone compositional and structural variables. In the case of spectral maps, points were considered independent replicates. The standard deviations of variables were used to depict the errors of the measurements. Differences among variables were considered significant at *p* < 0.05. All statistical analyses were performed using the *Origin Pro* (Microcal) software package.

## 3. Results   

### 3.1. Bone mineral characteristics and their relationship to mouse age   

**Figure 3:**
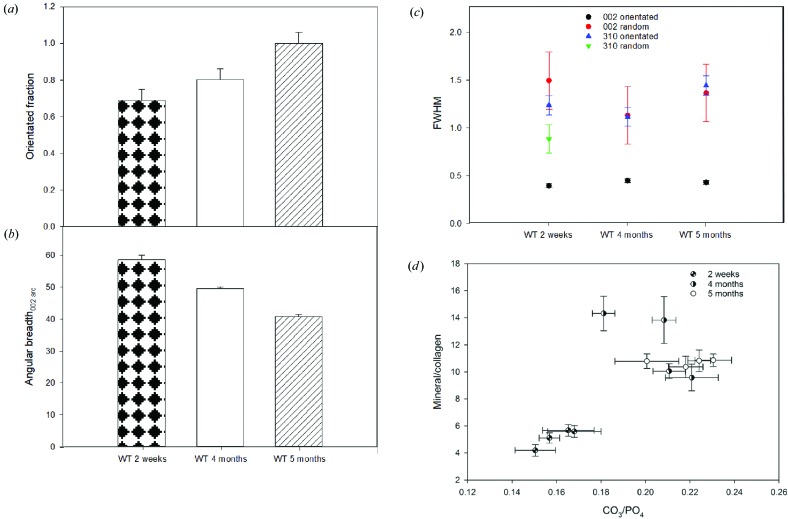
Changes in mineral characteristics related to age in WT mice. (*a*) The fraction of orientated bone crystals calculated using the 2D-XRD 002 ring intensity associated with oriented and random crystals for the two-week and four- or five-month-old WT mice. (*b*) The degree of crystal alignment measured using the change in 002 arc angular breadth. (*c*) The FWHM measured for oriented and random crystal populations using the 002 and 310 reflections in the 1D-XRD patterns. Error bars show the variation expected in the values based on multiple measurements of different pregnant mouse specimens. (*d*) Mineral-to-collagen versus carbonate-to-phosphate ratios obtained from individual Raman mapped areas in the three WT mouse specimens at different ages.

The X-ray diffraction data from tibiae cortical bone show that there are significant changes in the crystallinity and structural organization (crystal orientation) of bone crystals with age, genotype and health status of the mice. For instance, in the control group (WT virgin), the youngest mouse sample (WT_2w_v) showed the smallest FOBC, which correlated with the greatest degree of crystal misalignment [Figs. 3 ▸(*a*) and 3 ▸(*b*)]. As the age of the mice increased, the FOBC contribution also increased [Fig. 3 ▸(*a*)]. This is correlated with a decrease in the angular breadth of the 002 arc, which indicates that the alignment of the crystals increased with age [Fig. 3 ▸(*b*)]. As observed in the youngest mouse specimen, the FWHM_002_ for the FOBC in the four- and five-month-old mice showed a smaller value than the randomly oriented fraction [Fig. 3 ▸(*c*)], indicating that the FOBCs have a greater crystallinity. The FWHM_310_ for the FOBC did not change with age and was similar to that of the random fraction of the youngest mouse. This value could not be determined for the four- or five-month-old mice as the intensity was too low.

**Figure 4:**
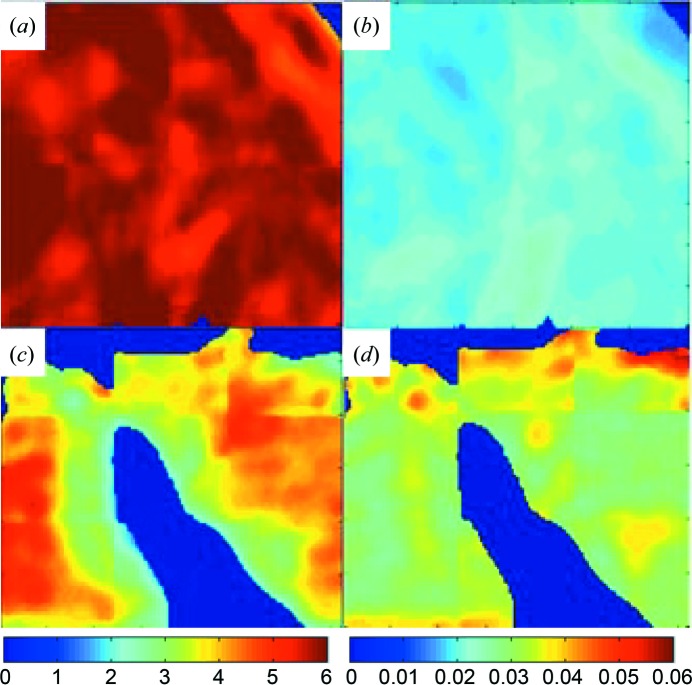
FT–IR transmission maps of femoral bones from (*a*), (*b*) virgin WT mouse femoral bones and (*c*), (*d*) virgin HYP mice. Panels (*a*) and (*c*) show the mineral-to-collagen ratio measured using the collagen 1600–1700 cm^−1^ bands and the phosphate bands spanning 900–1200 cm^−1^, respectively. Panels (*b*) and (*d*) show the variability in carbonate content of the bone mineral phase using the carbonate and phosphate spectral bands spanning 1414–1424 and 900–1200 cm^−1^, respectively. Each map is 100 × 120 µm.

Crystallinity was also evaluated using the FWHM of the symmetrical stretching band for phosphate at 960 cm^−1^, the most intense Raman band (see *e.g.* Fig. 1 ▸). The largest FWHM_960_ of the virgin WT mouse samples was found in the youngest mouse sample (16.05, s.d. 0.07 cm^−1^). In addition to a lower crystallinity, the Raman spectroscopy measurements of the youngest mouse also displayed a higher contribution of collagen to the spectra [Fig. 3 ▸(*d*)], measurable using the phosphate-to-1003 cm^−1^ ratio (Fig. S3). This is supported by the higher intensities of other collagen bands (Frushour & Koenig, 1975 ▸). Virgin mice analysed with FT–IR that were sacrificed at four (WT_4_v) and six months (WT_6_v) also had a higher mineral content (Table 2 ▸). The virgin mouse sacrificed at five months (WT_5_v) did not appear to follow this trend, with its mineral content slightly lower than the mouse sacrificed at four months. Analysis of the carbonate content and phosphate-to-collagen ratio from the FT–IR data for these specimens showed a homogeneous distribution [Fig. 4 ▸(*a*)]. This correlates with the low standard deviation obtained for these ratios using Raman spectroscopy, indicating that the femur and tibia are similar in our samples. Virgin WT mice show an increase in the carbonate ratio with age, from 0.163, s.d. 0.003, in the two-week-old mouse to 0.204, s.d. 0.005, in four-month-old and 0.221, s.d. 0.004, in five-month-old virgin mice measured using Raman spectroscopy. However, this effect is not visible in the FT–IR data (Table 2 ▸). Changes in crystallinity determined spectroscopically are correlated with changes in the carbonate content. Virgin WT mice show a homogeneous distribution of carbonate content and phosphate-to-collagen ratio when they have reached five months, as shown in the FT–IR maps in Fig. 4 ▸.

### 3.2. Effect of different health conditions on bone mineral characteristics   

**Figure 5:**
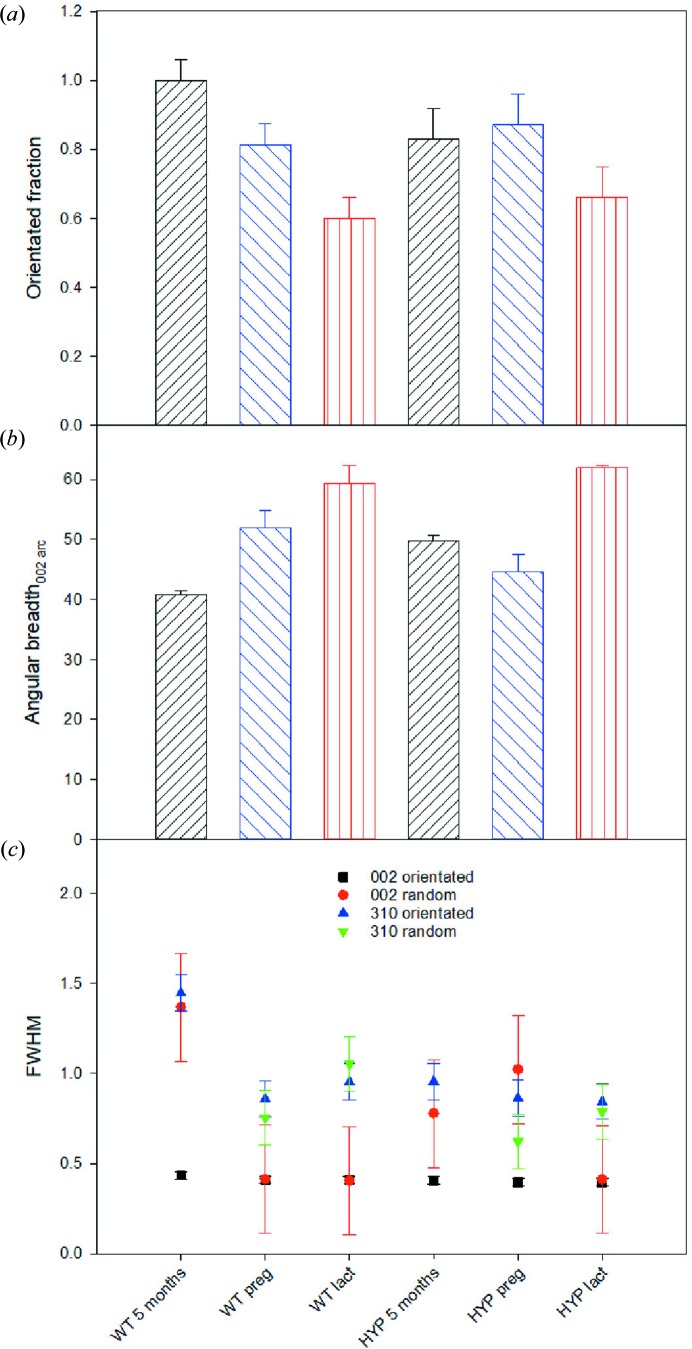
Changes in mineral characteristics due to increased remodelling related to pregnancy and lactation in WT and HYP mice. (*a*) The FOBC in the bone specimens, calculated using the 002 ring intensity associated with oriented and random crystals. (*b*) The angular breadth of the 002 arc from 2D-XRD patterns, indicating the degree of crystal alignment with the long axis of the bone specimen. (*c*) FWHM measured from the 002 and 310 reflections in the 1D-XRD patterns for oriented and random crystal populations. Error bars show the variation expected in the values based on multiple measurements of different pregnant mouse specimens.

Bone mineral in pregnant and especially lactating mice had a smaller FOBC compared with virgin mice [Fig. 5 ▸(*a*)]. As observed with the age-related samples, a decrease in the FOBC is coupled with an increase in the angular breadth of the 002 arcs, indicating an increase in the misalignment of the crystals [Fig. 5 ▸(*b*)]. In general, we observed the following trend of decreasing FOBC and crystal orientation: virgin > pregnant > lactating. The FWHM_002_ displayed no change for the FOBC between the five-month-old virgin, pregnant and lactating WT mice. However, in the pregnant and lactating WT mice there was a large decrease in the FWHM of randomly oriented crystals [Fig. 5 ▸(*c*)], implying a diffracting domain size that is similar to that found in the FOBC. There was also a decrease in the FWHM_310_ in the FOBC in the pregnant and lactating WT mice. Again, crystallinity decreases according to the following sequence: virgin > pregnant > lactating.

The Raman spectroscopy data show that the phosphate-to-1003 cm^−1^ ratios increased from 10.7, s.d. 0.2, in the virgin mouse to 29.1, s.d. 0.6, in the pregnant mice, before returning to lower ratios of 14.1, s.d. 0.6, in the lactating mouse. This is consistent with the FT–IR data, which showed almost no change between the collagen content of the virgin WT and lactating WT mice (Table 2 ▸). Despite the increase in diffracting domain size, indicating an increase in long-range crystallinity, the Raman spectroscopy FWHM_960_ measurements suggested that short-range crystallinity decreased for the pregnant (15.70, s.d. 0.03 cm^−1^) and lactating (15.52, s.d. 0.04 cm^−1^) mice compared with the virgin mouse (15.42, s.d. 0.04 cm^−1^). No change in the carbonate content was detected in the vibrational spectroscopy analyses related to remodelling regime.

### 3.3. Evidence for mineral changes due to hypophosphatemia   

The X-ray diffraction data show that bone mineral in the virgin HYP mouse had a smaller FOBC than that in the virgin WT mouse [Fig. 5 ▸(*a*)]. Also, there is, as expected, an increase in the angular breadth of the 002 arc [Fig. 5 ▸(*b*)]. The FWHM_002_ for the FOBC did not differ between WT and HYP mice. However, the FWHM_002_ for the randomly oriented fraction in HYP mice was smaller than that for the virgin WT mice [Fig. 5 ▸(*c*)], indicating that the crystals had larger domains along the *c* axis in the virgin HYP mouse. Similarly, values of the FWHM_310_ were smaller in the HYP virgin mouse. The crystallinity obtained from Raman spectroscopic analysis was in agreement with the 2D-XRD data, where the FWHM_960_ of the virgin HYP mouse (16.06, s.d. 0.06) was higher than that of the virgin WT mouse (15.42, s.d. 0.04). The virgin HYP mouse also showed a higher variability of its phosphate-to-collagen ratio compared with the virgin WT mouse, as can be observed by comparing Figs. 4 ▸(*a*) and 4 ▸(*c*). This is reflected in the Raman spectroscopy data and standard deviation, which showed an increase in the phosphate-to-1003 cm^−1^ ratio from 10.7, s.d. 0.2, in the virgin WT mouse to 12.6, s.d. 0.7, in the virgin HYP mouse. The FT–IR map demonstrated that the carbon­ate content within the virgin HYP mouse bone sections had a higher variability than those from virgin WT mice [*c.f.* Figs. 4 ▸(*b*) and 4 ▸(*d*)]. A higher average carbonate content for the virgin HYP mouse compared with the virgin WT mice was also determined from Raman spectroscopic measurements, where the virgin HYP mouse had a carbonate-to-phosphate ratio of 0.26, s.d. 0.01, whereas the virgin WT mouse had a ratio of 0.221, s.d. 0.004.

For HYP mice, the lactating HYP mouse showed the greatest crystal misalignment [Fig. 5 ▸(*b*)]. The pregnant HYP mouse measurements did not follow the expected trend observed in the WT mice for FOBC: virgin > pregnant > lactating. Instead, the pregnant HYP mice have a similar to slightly elevated FOBC and slightly lower 002 angular breadth compared with the virgin HYP mouse. As observed in the WT mice, the lactating HYP mouse FWHM_002_ measurement was the same for both the randomly oriented and oriented fraction. Unlike the WT mice, however, the FWHM of the 310 reflection showed no difference from that of the HYP virgin mouse, indicating that no change in domain size of 310 occurred in relation to remodelling during lactation. The unusual behaviour of the angular breadth and oriented fraction in pregnant HYP mice was mirrored by an increase in the FWHM_002_ of the randomly oriented fraction. The FWHM_310_ of the FOBC displayed similar values independent of the health status (pregnant, lactating) or remodelling regime in the HYP mice.

Regarding the Raman data, the lactating HYP mouse showed the highest FWHM_960_ measured in this study (16.59, s.d. 0.13), consistent with the lower long-range crystallinity determined by 2D-XRD [Fig. 5 ▸(*c*)]. Also, the pregnant HYP mice show a similar FWHM_960_ on average to the virgin HYP mouse in the Raman analysis (16.02, s.d. 0.01, and 16.06, s.d. 0.06, respectively), in agreement with the 2D-XRD data that there is minimal change in the mineral crystallinity characteristics between the pregnant and virgin HYP mice. Higher phosphate-to-collagen ratios are observed in the virgin HYP mouse (HYP_5_v) compared with the lactating HYP mouse (HYP_5_l) in the FT–IR analysis. The Raman spectroscopy analysis showed that the phosphate-to-1003 cm^−1^ ratio for the lactating HYP mouse was the same as that observed in the pregnant HYP mouse, 25.5, s.d. 2.2, and 25.3, s.d. 1.9, respectively.

The carbonate content in the HYP mice increased following the sequence virgin (0.26, s.d. 0.01) < pregnant (0.30, s.d. 0.02) < lactating (0.34, s.d. 0.01). These results are consistent with the FT–IR analysis, which showed a high variability of the carbonate content within the HYP bones and a higher level in the lactating mice compared with the virgin HYP mouse. The amount of substituted carbonate within the mineral phase was estimated using the regression line shown in Fig. S1. Calculations of carbonate content showed that the most carbonated sample, the lactating HYP mouse, had a carbonate content of 8.86 (s.d. 0.01) wt%. In contrast, the lactating WT mouse has a carbonate content of 5.85 (s.d. 0.01) wt%. These values are within the ranges observed previously for bovine (Awonusi *et al.*, 2007 ▸) and rabbit (Penel *et al.*, 1998 ▸) bones, which have been shown to contain up to 9 wt% carbonate.

Finally, analysis of variance (two-way ANOVA) was used to analyse whether the genotypes and health status determine the observed changes in bone properties. The 2D-XRD data show that there are significant differences between mouse genotypes for the FWHM of the 002 reflection of the FOBC (WT and HYP; *p* = 0.016) but not among mice with different health status (*p* = 0.234). The 002 arc angular breadth showed no significant differences between mouse genotypes (WT and HYP; *p* = 0.50) but here was a significant difference among mice with different health status (*p* = 0.027). However, the FOBC decreases following the same sequence but the differences are not statistically significant (*p* = 0.102). The Raman data show that there are highly significant differences in bone mineral carbonate content, crystallinity and phosphate-to-1003 cm^−1^ band ratio between WT and HYP mice (*p* < 0.001). Also, there are significant differences for the same parameters among mice with different health status (virgin, pregnant, lactating) that represent increasing bone remodelling rate regimes (*p* < 0.001). The FT–IR carbonate content only shows a significant difference between WT and HYP mice (*p* < 0.001) but not among the different health status mice analysed (virgin and lactating; *p* = 0.695). The degree of mineralization did not show any significant difference between mice with different genotypes or health status (*p* > 0.2).

## 4. Discussion   

### 4.1. Correlation between degree of bone maturation, mineral orientation and chemistry   

The lower mineral crystallinity and greater disorder indicated in the XRD analysis from the youngest sample (WT_2w_v) is consistent with newly deposited bone (Ishimoto *et al.*, 2013 ▸), as the rate of bone turnover is expected to be highest in the youngest mouse (Price *et al.*, 2005 ▸). Thus, the mineral population sampled will have a greater proportion of less mature mineral crystals (Rey *et al.*, 1991 ▸). Similarly, the increasing values for oriented fraction and alignment of the four- and five-month-old mice (Fig. 3 ▸) fit with bone mineral maturation as average tissue age increases. The mineral crystal sizes, as calculated from the XRD measurements (Table S1), are similar to those documented previously in chicken (Bonar *et al.*, 1983 ▸), bovine (Kuhn *et al.*, 2008 ▸) and rat (Turunen *et al.*, 2011 ▸) specimens. Bone mineral shows highly anisotropic peak broadening for the 002 and 310 reflections as the crystallites are elongated along the *c* axis ([001] direction; Fratzl *et al.*, 2004 ▸). In addition, the calculated crystallite sizes are smaller in the randomly oriented fraction, consistent with less-mature newly deposited minerals. This is also reflected in the short-range crystallinity of the mineral phases, measured from the Raman spectra, which increases with increasing age. Previous vibrational spectroscopy work on rabbit bones has shown that younger animals have a lower carbonate content within the mineral structure of the crystallites (Turunen *et al.*, 2011 ▸). Previous work with FT–IR and WT mice showed the same trend (Boskey *et al.*, 1998 ▸). Our results from Raman spectroscopy analysis also show this progression in bone mineral maturation. Although the structural variations observed with XRD continued to change above four months of age, the chemical variations observed in our specimens were minimal.

Bone is an effective store of phosphate and Ca that can be readily used by the body during times of high demand for these components (Glimcher, 1998 ▸). In normal mice, the demand for Ca and phosphate for accretion of mineral in the developing foetus is predominantly compensated for by increased uptake of these components from food sources. A decrease in parathyroid hormone (PTH) was observed in the pregnant WT mice that we studied (Macica *et al.*, 2016 ▸), which is thought to protect the maternal skeleton from remodelling (Turner *et al.*, 1988 ▸). Similarly, increased intestinal uptake is expected in the normal mice as upregulation of 1,25-di­hydroxy­vitamin D was also observed in the pregnant mice studied here (Macica *et al.*, 2016 ▸). Therefore, limited remodelling of the bone is expected in the normal mice. However, the XRD measurements on the pregnant mice indicate that the mineral crystal population is not similar to that found in the virgin mouse as the calculated crystal size increases in the randomly oriented crystals. Changes in the carbonate content of the mineral could explain the changes in calculated crystal size (Zapanta-LeGeros, 1965 ▸), but no changes in carbonate content were observed in the vibrational spectroscopy data. In the pregnant mice there is evidence for a decrease in the short-range crystallinity corresponding to an increased phosphate-to-1003 cm^−1^ ratio. Increased remodelling should produce lower crystal-to-collagen ratios, and therefore the increased phosphate-to-1003 cm^−1^ ratio could indicate an increase in the HPO_4_ concentrations in the initial mineral phase produced in the higher remodelling regime in the pregnant mice. This is consistent with a more immature population being sampled, as these crystals have been shown to have high HPO_4_ contents in rats and bovine samples (Legros *et al.*, 1987 ▸). The change in chemistry indicates that mineral deposition was occurring during pregnancy in the mouse samples. Indeed, in normal mouse bone the degree of mineralization can increase by 5–10% during pregnancy (Sharpe *et al.*, 2003 ▸; Woodrow *et al.*, 2003 ▸). However, elevated levels of the type-1 collagen-degrading enzyme MMP-13 and tartrate-resistant acid phosphatase (TRAP) in the pregnant WT mice (Macica *et al.*, 2016 ▸) indicate that resorption is already occurring in these animals, probably due to the time of sacrifice, which was post partum and thus the onset of lactation.

Unlike pregnancy, during lactation bone becomes the dominant source of Ca, where increased bone remodelling can result in up to 30% mineral loss in normal mice within 21 days (Sharpe *et al.*, 2003 ▸). The largest loss is typically observed in trabecular bone, but increased levels of MMP-13 in the cortical bone of the normal mice studied here also suggest there was increased cortical remodelling (Macica *et al.*, 2016 ▸). Thus, the changes in bone mineral crystal-orientation characteristics measured using 2D-XRD can be linked with higher levels of remodelling in the pregnant and lactating WT mice. This is consistent with the progressive decrease in the FOBC from virgin > pregnant > lactating and the concomitant increase in the angular breadth of the 002 arc. Hence, the XRD analysis shows that more newly deposited immature bone mineral populations are present in the lactating mice. Thus, changes in bone mineral chemistry, crystallinity and crystal orientation are mainly due to the rate of bone turnover.

### 4.2. Evidence for changes in bone mineral characteristics due to hypophosphatemia   

HYP mice have been shown to have an impaired uptake of Ca (Macica *et al.*, 2016 ▸) and increased phosphate excretion (Liang *et al.*, 2009 ▸), and hence lower levels of blood serum Ca and P compared with their WT mouse counterparts (Macica *et al.*, 2016 ▸). In addition to an impaired ability to mineralize bone, this suggests that the mineralization solution has lower levels of Ca and P. Evidence for higher levels of bone remodelling are also implied in the HYP mouse, as blood serum levels of PTH are elevated in comparison with normal mice (Macica *et al.*, 2016 ▸). However, HYP mice have decreased numbers of osteoclasts (Hayashibara *et al.*, 2007 ▸) and lower osteoclast activity (Ono *et al.*, 1996 ▸). Higher levels of MMP-13 within the cortical bone of virgin HYP mice, as well as its association with osteocyte lacunae (Macica *et al.*, 2016 ▸), suggest that alternative forms of bone remodelling through osteocyte activity may be required to mobilize mineral in these mice (Wysolmerski, 2012 ▸). A higher level of cortical bone remodelling agrees with our observation of lower FOBC and alignment in the virgin HYP mouse compared with the virgin WT mouse at the same age. In the FT–IR maps it can be seen that the mineral-to-collagen ratios are more heterogeneous in the virgin HYP mouse throughout the cortical bone in comparison with the WT mouse. This is also reflected in observations from HYP mouse bone histology studies, which show areas of cortical bone with apparent ‘porosity’ in high-resolution micro-computed tomography images that are related to unmineralized collagen and a decrease in the mineral content (Macica *et al.*, 2016 ▸).

The carbonate content in the virgin HYP mouse is also more variable than in the WT mouse, and the overall mineral carbonate content measured in the FT–IR maps is higher (Table 2 ▸). This is reflected in the Raman spectroscopic analysis. As the Raman spectroscopy data are expected to probe the carbonate substituted into the apatite mineral structure (Penel *et al.*, 1998 ▸), while the FT–IR bands probe the overall mineral carbonate content (both apatitic and non-apatitic environments) (Combes *et al.*, 2016 ▸), the elevation of carbonate observed with both these techniques indicates that there is a higher amount of carbonate substitution into the mineral in the HYP mouse bones. This is the opposite of what would be expected for higher remodelling rates based on the Raman spectroscopy findings from the WT_2w_v mouse and FT–IR analysis from ageing WT mice (Boskey *et al.*, 1998 ▸). Increased carbonate content is associated with a decrease in the calculated crystallite size for the 310 reflection of randomly oriented crystals within the virgin HYP mouse and a decrease in short-range crystallinity observed in the Raman spectroscopy data. Broadening of the XRD 002 peak with increasing carbonate content in synthetic hy­droxy­apatites has been shown to correlate directly with increases in the solubility of the mineral phase (Baig *et al.*, 1996 ▸). If this trend holds true for the chemical composition of the HYP mouse bone crystals, this implies that the mature FOBC should have a similar solubility to those found in the WT mice. In contrast, the decrease in the FWHM_002_ of the HYP mouse randomly oriented crystal fraction implies that these crystals will have a lower solubility in comparison with their counterparts in the WT mouse, despite an apparent increase in carbonate and decrease in short-range crystallinity.

HYP mice were shown to deploy similar adaptations to those of WT mice to meet the demands of Ca and P during pregnancy and lactation. This includes elevation of the levels of MMP-13 and TRAP (Macica *et al.*, 2016 ▸), demonstrating that these bones have a higher degree of remodelling than the virgin HYP mouse. In HYP mice this is related to a higher carbonate content compared with both the virgin HYP mouse and the WT counterparts. In addition, the 1003 cm^−1^ band in the pregnant and lactating HYP mice is higher, without a concomitant decrease in the mineral-to-collagen ratio. This suggests that, similar to the WT mice, the bone mineral deposited in HYP mice during pregnancy and lactation also has a higher HPO_4_ component. As discussed above, newly deposited bone mineral is expected to be poorly organized with random crystal orientations (Ishimoto *et al.*, 2013 ▸), but a similar crystallite alignment was found between the pregnant and virgin HYP mice, as well as similar crystallinity in both long- and short-range ordering. Previous observations of bone mineral maturation (Rey *et al.*, 1991 ▸) and synthetic nanoparticles (Cazalbou *et al.*, 2004 ▸) show an increase in crystallinity and carbonate with time compared with the initially precipitated material. Thus, the bone mineral measured in the pregnant HYP mice is expected to be dominated by more mature crystallites than those observed in the HYP lactating and virgin mice. In contrast, although cortical bone remodelling appears to remain high in the lactating HYP mouse, the alignment and short-range order are lowered. Taken together with the increased carbonate content of the mineral, this suggests that remineralization of the bone tissue with a higher carbonate content occurs during lactation, but appears to be limited during pregnancy.

## 5. Conclusions   

A synergistic approach to characterizing bone that unites 2D-XRD and vibrational spectroscopy has provided an unparalleled insight into the changes in bone mineral structure and chemistry associated with changes in the rate of bone re­modelling and deficient mineralization. Changes in the remodelling regime produced complicated signatures in the bone mineral structural organization that can only be un­ravelled using our approach. The information about unit-cell size gained from XRD can be used to clarify potential sources of changing intensity in the vibrational spectroscopy data. For example, both the HYP and WT mice show evidence for a potential increase in the HPO_4_ content of the bone mineral during pregnancy and lactation, reflecting the higher rates of bone turnover observed using other markers (Macica *et al.*, 2016 ▸). The increased osteocyte/MMP-13 activity and increased bone mineral carbonate content observed previously (Macica *et al.*, 2016 ▸) are also supported by the decrease in crystallinity and crystal alignment observed in the HYP mice. However, we could only analyse a small number of samples, so these trends should be verified with more extensive studies that also include the orientation of the collagen, *e.g.* through examination of the bone samples prior to embedding in epoxy. This will also allow the mechanical properties of the bone to be tested, as changes in the mineralization extent and mineral quality within HYP mouse bones has been linked to increasing bone fragility (Camacho *et al.*, 1995 ▸).

Lower levels of Ca, and particularly of phosphate, in the HYP mice can be correlated with a clear chemical change in the bone mineral in our samples, where lower phosphate is partially compensated by the incorporation of additional carbonate during mineral growth. The limited sample size studied here indicates that HYP mice produce bone mineral with a lower short-range crystallinity consistent with higher apatitic and non-apatitic carbonate, as well as a higher degree of crystal misorientation within the bone. Synthetic *B*-site carbonated apatites show a higher solubility with increasing carbonate incorporation (Baig *et al.*, 1996 ▸). However, the XRD measurements from the HYP mice indicate that increased carbonate in bone, as detected using vibrational spectroscopy, may not be directly correlated with an increase in mineral solubility.

## Supplementary Material

Additional tables and figures. DOI: 10.1107/S1600576719009361/jo5049sup1.pdf

## Figures and Tables

**Table 1:** Descriptions of the mouse samples examined WT refers to wild-type mice and HYP denotes mice with a genetic disorder that results in hypophosphatemia.

Sample number†	Specimen	No. of specimens	Age at sacrifice (months)	Condition	Lactation duration	Analytical method
WT_2w_v	WT	1	2 weeks	Virgin		Raman, 2D-XRD
WT_3_v	WT	1	3	Virgin		IR
HYP_3_l	HYP	1	3	Lactating	1 week	IR
WT_4_v	WT	1	4	Virgin		IR, Raman, 2D-XRD
WT_4_l	WT	1	4	Lactating	3 weeks	IR, 2D-XRD
WT_5_v‡	WT	1	5	Virgin		IR, Raman, 2D-XRD
HYP_5_v‡	HYP	1	5	Virgin		Raman, 2D-XRD
HYP_5_v2‡	HYP	1	5	Virgin		IR, 2D-XRD
WT_5_p*n* ‡	WT	3	5	Pregnant		Raman, 2D-XRD
HYP_5_p*n* ‡	HYP	3	5	Pregnant		Raman, 2D-XRD
WT_5_l‡	WT	1	5	Lactating	3 weeks	Raman, 2D-XRD
HYP_5_l‡	HYP	1	5	Lactating	3 weeks	IR, Raman, 2D-XRD

† Sample names indicate the genotype (WT or HYP) followed by the age at sacrifice in months (with the exception of 2w which denotes 2 weeks), the remodelling regime (v = virgin, p = pregnant, l = lactating) and additional information, where *n* denotes different numbers related to the multiple samples that were examined and 2w or 3w indicate different lactation periods of 2 and 3 weeks, respectively.

‡ Samples also studied by Macica *et al.* (2016 ▸).

**Table 2:** Carbonate-to-phosphate and mineral-to-collagen ratios calculated from the averages from the individual FT–IR spectra within the mapped areas Errors are the standard deviation (1σ) of the data.

Sample	Carbonate/phosphate	Mineral/collagen
WT_3_v	0.021 ± 0.003	5.2 ± 0.7
HYP_3_l	0.027 ± 0.004	4.4 ± 0.6
WT_4_v	0.022 ± 0.004	6.0 ± 0.6
WT_4_l2w	0.022 ± 0.004	4.9 ± 0.9
WT_5_v	0.024 ± 0.001	5.6 ± 0.7
HYP_5_v2	0.028 ± 0.004	4.9 ± 0.9
HYP_5_l	0.030 ± 0.002	4.2 ± 0.8

## References

[bb1] Acerbo, A. S., Carr, G. L., Judex, S. & Miller, L. M. (2012). *Anal. Chem.* **84**, 3607–3613. 10.1021/ac203375d PMC3364542 22455306

[bb2] Amenta, E., King, H. E., Petermann, H., Uskoković, V., Tommasini, S. M. & Macica, C. M. (2018). *Ther. Adv. Chron. Dis.* **9**, 268–281. 10.1177/2040622318804753 PMC6348532 30719271

[bb3] Awonusi, A., Morris, M. D. & Tecklenburg, M. M. (2007). *Calcif. Tissue Int.* **81**, 46–52. 10.1007/s00223-007-9034-0 17551767

[bb4] Baig, A. A., Fox, J. L., Hsu, J., Wang, Z., Otsuka, M., Higuchi, W. I. & LeGeros, R. Z. (1996). *J. Colloid Interface Sci.* **179**, 608–617.

[bb5] Bala, Y., Farlay, D., Delmas, P. D., Meunier, P. J. & Boivin, G. (2010). *Bone*, **46**, 1204–1212. 10.1016/j.bone.2009.11.032 19969115

[bb6] Bazin, D., Chappard, C., Combes, C., Carpentier, X., Rouzière, S., André, G., Matzen, G., Allix, M., Thiaudière, D., Reguer, S., Jungers, P. & Daudon, M. (2009). *Osteoporos. Int.* **20**, 1065–1075. 10.1007/s00198-009-0868-3 19340497

[bb7] Boivin, G. & Meunier, P. J. (2002). *Calcif. Tissue Int.* **70**, 503–511. 10.1007/s00223-001-2048-0 12019458

[bb8] Bonar, L. C., Roufosse, A. H., Sabine, W. K., Grynpas, M. D. & Glimcher, M. J. (1983). *Calcif. Tissue Int.* **35**, 202–209. 10.1007/BF02405032 6850400

[bb9] Boskey, A. L. (2002). *J. Musculoskelet. Neuron Interact.* **2**, 532–534. 15758386

[bb10] Boskey, A. L., Gadaleta, S., Gundberg, C., Doty, S. B., Ducy, P. & Karsenty, G. (1998). *Bone*, **23**, 187–196. 10.1016/s8756-3282(98)00092-1 9737340

[bb11] Busa, B., Miller, L. M., Rubin, C. T., Qin, Y. & Judex, S. (2005). *Calcif. Tissue Int.* **77**, 386–394. 10.1007/s00223-005-0148-y 16362460

[bb12] Camacho, N. P., Rimnac, C. M., Meyer, R. A. Jr, Doty, S. & Boskey, A. L. (1995). *Bone*, **17**, 271–278. 10.1016/8756-3282(95)00210-5 8541141

[bb13] Carpenter, T. O., Imel, E. A., Holm, I. A., Jan de Beur, S. M. & Insogna, K. L. (2011). *J. Bone Miner. Res.* **26**, 1381–1388. 10.1002/jbmr.340 PMC3157040 21538511

[bb14] Cazalbou, S., Combes, C., Eichert, D., Rey, C. & Glimcher, M. J. (2004). *J. Bone Miner. Metab.* **22**, 310–317. 10.1007/s00774-004-0488-0 15221488

[bb15] Combes, C., Cazalbou, S. & Rey, C. (2016). *Minerals*, **6**, 34.

[bb16] Dominguez-Gasca, N., Benavides-Reyes, C., Sánchez-Rodríguez, E. & Rodríguez-Navarro, A. B. (2019). *Eur. J. Mineral.* **31**, 209–216.

[bb17] Fratzl, P., Gupta, H. S., Paschalis, E. P. & Roschger, P. (2004). *J. Mater. Chem.* **14**, 2115–2123.

[bb18] Frushour, B. G. & Koenig, J. L. (1975). *Biopolymers*, **14**, 379–391. 10.1002/bip.1975.360140211 1174668

[bb19] Glimcher, M. J. (1998). *Metabolic Bone Disease and Clinically Related Disorders*, 3rd ed., ch. 2, pp. 23–50. New York: Academic Press.

[bb20] Glimcher, M. J. (2006). *Rev. Mineral. Geochem.* **64**, 223–282.

[bb21] Hayashibara, T., Hiraga, T., Sugita, A., Wang, L., Hata, K., Ooshima, T. & Yoneda, T. (2007). *J. Bone Miner. Res.* **22**, 1743–1751. 10.1359/jbmr.070709 17638577

[bb22] Ishimoto, T., Nakano, T., Umakoshi, Y., Yamamoto, M. & Tabata, Y. (2013). *J. Bone Miner. Res.* **28**, 1170–1179. 10.1002/jbmr.1825 23184575

[bb23] Kuhn, L. T., Grynpas, M. D., Rey, C. C., Wu, Y., Ackerman, J. L. & Glimcher, M. J. (2008). *Calcif. Tissue Int.* **83**, 146–154. 10.1007/s00223-008-9164-z PMC6181642 18685796

[bb24] Langford, J. I. & Wilson, A. J. C. (1978). *J. Appl. Cryst.* **11**, 102–113.

[bb25] Legros, R., Balmain, N. & Bonel, G. (1987). *Calcif. Tissue Int.* **41**, 137–144. 10.1007/BF02563793 3117340

[bb26] Liang, G., Katz, L. D., Insogna, K. L., Carpenter, T. O. & Macica, C. M. (2009). *Calcif. Tissue Int.* **85**, 235–246. 10.1007/s00223-009-9270-6 PMC2988401 19609735

[bb27] Londoño-Restrepo, S. M., Zubieta-Otero, L. F., Jeronimo-Cruz, R., Mondragon, M. A. & Rodrigues-Garcia, M. E. (2019). *J. Raman Spectrosc.* **50**, 1120–1129.

[bb28] Macica, C. M., King, H. E., Wang, M., McEachon, C. L., Skinner, C. W. & Tommasini, S. M. (2016). *Bone*, **85**, 59–69. 10.1016/j.bone.2015.12.056 PMC7429445 26825813

[bb29] McElderry, J. P., Zhu, P., Mroue, K. H., Xu, J., Pavan, B., Fang, M., Zhao, G., McNerny, E., Kohn, D. H., Franceschi, R. T., Holl, M. M. B., Tecklenburg, M. M. J., Ramamoorthy, A. & Morris, M. D. (2013). *J. Solid State Chem.* **206**, 192–198. 10.1016/j.jssc.2013.08.011 PMC3835554 24273344

[bb30] Nakamura, M., Hiratai, R., Hentunen, T., Salonen, J. & Yamashita, K. (2016). *ACS Biomater. Sci. Eng.* **2**, 259–267. 10.1021/acsbiomaterials.5b00509 33418638

[bb31] Ono, T., Tanaka, H., Yamate, T., Nagai, Y., Nakamura, T. & Seino, Y. (1996). *Endocrinology*, **137**, 2633–2637. 10.1210/endo.137.6.8641218 8641218

[bb32] Pasteris, J. D. & Ding, D. Y. (2009). *Am. Mineral.* **94**, 53–63.

[bb33] Penel, G., Leroy, G., Rey, C. & Bres, E. (1998). *Calcif. Tissue Int.* **63**, 475–481. 10.1007/s002239900561 9817941

[bb34] Price, C., Herman, B. C., Lufkin, T., Goldman, H. M. & Jepsen, K. J. (2005). *J. Bone Miner. Res.* **20**, 1983–1991. 10.1359/JBMR.050707 16234972

[bb35] Querido, W., Ailavajhala, R., Padalkar, M. & Pleshko, N. (2018). *Appl. Spectrosc.* **72**, 1581–1593. 10.1177/0003702818789165 29972319

[bb36] Rey, C., Combes, C., Drouet, C. & Glimcher, M. J. (2009). *Osteoporos. Int.* **20**, 1013–1021. 10.1007/s00198-009-0860-y PMC2760485 19340505

[bb37] Rey, C., Lian, J., Grynpas, M., Shapiro, F., Zylberberg, L. & Glimcher, M. J. (1989). *Connect. Tissue Res.* **21**, 267–273. 10.3109/03008208909050016 2605951

[bb38] Rey, C., Renugopalakrishman, V., Collins, B. & Glimcher, M. J. (1991). *Calcif. Tissue Int.* **49**, 251–258. 10.1007/BF02556214 1760769

[bb39] Rodriguez-Navarro, A. B. (2006). *J. Appl. Cryst.* **39**, 905–909.

[bb40] Rodriguez-Navarro, A. B., McCormack, H. M., Fleming, R. H., Alvarez-Lloret, P., Romero-Pastor, J., Dominguez-Gasca, N., Prozorov, T. & Dunn, I. C. (2018). *J. Struct. Biol.* **201**, 36–45. 10.1016/j.jsb.2017.10.011 29109023

[bb41] Rollin-Martinet, S., Navrotsky, A., Champion, E., Grossin, D. & Drouet, C. (2013). *Am. Mineral.* **98**, 2037–2045.

[bb42] Sharpe, C. J., Fudge, N. J. & Kovacs, C. S. (2003). *Bone Conf. Proc.* **32**, S227.

[bb43] Turner, M., Barré, P. E., Benjamin, A., Goltzman, D. & Gascon-Barré, M. (1988). *Miner. Electrolyte Metab.* **14**, 246–252. 3211093

[bb44] Turunen, M. J., Saarakkala, S., Rieppo, L., Helminen, H. J., Jurvelin, J. S. & Isaksson, H. (2011). *Appl. Spectrosc.* **65**, 595–603. 10.1366/10-06193 21639980

[bb45] Weiner, S. & Wagner, H. D. (1998). *Annu. Rev. Mater. Res.* **28**, 271–298.

[bb46] Wenk, H. & Heidelbach, F. (1999). *Bone*, **24**, 361–369. 10.1016/s8756-3282(98)00192-6 10221548

[bb47] Wojdyr, M. (2010). *J. Appl. Cryst.* **43**, 1126–1128.

[bb48] Woodrow, J. P., Noseworthy, C. S., Fudge, N. J., Hoff, A. O., Gagel, R. F. & Kovacs, C. S. (2003). *Am. Soc. Bone Miner. Res. Conf.* **18**, S37.

[bb49] Wopenka, B. & Pasteris, J. D. (2005). *Mater. Sci. Eng. C*, **25**, 131–143.

[bb50] Wysolmerski, J. J. (2012). *BoneKEy Rep.* **1**, 229. 10.1038/bonekey.2012.229 PMC3868715 24363929

[bb51] Yoder, C., Pasteris, J., Worcester, K., Schermerhorn, D., Sternlieb, M., Goldenberg, J. & Wilt, Z. (2012). *Minerals*, **2**, 100–117.

[bb52] Zapanta-LeGeros, R. (1965). *Nature*, **206**, 403–404. 10.1038/206403a0 5835710

[bb53] Ziv, V. & Weiner, S. (1994). *Connect. Tissue Res.* **30**, 165–175. 10.3109/03008209409061969 8039384

