# Supplementary material for: Correlative vibrational spectroscopy and 2D X-ray diffraction to probe the mineralization of bone in phosphate-deficient mice
Source: J Appl Crystallogr. 2019 Aug 23;52(Pt 5):960–71. doi: 10.1107/S1600576719009361 (PMC6782074; doi:10.1107/S1600576719009361)
Supplement: Supplementary file 1 [file j-52-00960-sup1.pdf]

JOURNAL OF
APPLIED
CRYSTALLOGRAPHY

Volume 52 (2019)

Supporting information for article:

Correlative vibrational spectroscopy and 2D X-ray diffraction to probe the mineralization of bone in phosphate-deficient mice

Helen E. King, Steven M. Tommasini, Alejandro B. Rodriguez-Navarro, Brandon Q. Mercado and H. Catherine W. Skinner

The data used in this study can be found at the Pangaea online data repository, data issue identifier PDI 15212.

Table S1 Full width at half maximum (FWHM) from the phosphate symmetrical stretch Raman band and 2D-XRD 2 theta scans for 002 and 310 reflections from mature crystals in the bone samples, see text for full description. Length parameters (L) calculated from XRD data using Scherrer equation assuming that the strain factor is negligible. Errors in the FWHM measurement are around 0.003° based on fitting the same spectrum multiple times.

**Sample**	**$FWHM_{960}$ (cm^{-1})**	**$FWHM_{002}$ (°)**	**$FWHM_{310}$(°)**	**L_{002}(nm)**	**L_{310}(nm)**
WT_2w_v	16.05 ±0.07	0.484	0.874	20.8 ±1.0	6.9 ±0.3
WT_4_v	15.85 ±0.05	0.497	0.834	18.4 ±0.9	7.5 ±0.3
WT_4_l2w	15.59 ±0.03	0.441	0.762	20.7 ±1.1	10.8 ±0.4
WT_4_l3w	-	0.501	0.763	18.8 ±0.9	9.6 ±0.4
WT_5_v	15.42 ±0.04	0.514	0.841	19.2 ±0.9	5.8 ±0.3
HYP_5_v	16.06 ±0.06	0.492	0.734	20.9 ±1.0	9.1 ±0.4
HYP_5_v2	-	0.430	0.696	20.4 ±1.1	11.9 ±0.4
WT_5_p2	15.76 ±0.06	0.425	0.843	21.4 ±1.1	9.1 ±0.3
WT_5_p3	15.69 ±0.03	0.457	0.754	20.7 ±1.0	10.7 ±0.4
WT_5_p5	15.70 ±0.04	0.434	0.748	20.5 ±1.1	10.5 ±0.4
WT_5_p7	-	0.454	0.741	19.9 ±1.0	10.7 ±0.4
WT_5_p8	-	0.478	0.773	20.3 ±1.0	9.2 ±0.4
HYP_5_p3	16.10 ±0.1	0.462	0.781	20.2 ±1.0	9.3 ±0.4
HYP_5_p4	16.01 ±0.07	0.474	0.763	21.0 ±1.0	10.3 ±0.4
HYP_5_p5	15.95 ±0.1	0.498	0.783	20.8 ±1.0	10.3 ±0.4
HYP_5_p6	-	0.490	0.770	22.4 ±1.1	8.4 ±0.4
HYP_5_p7	-	0.443	0.762	21.7 ±1.1	11.4 ±0.4
WT_5_l	15.52 ±0.04	0.508	0.792	20.5 ±0.9	9.0 ±0.3
HYP_5_l	16.59 ±0.13	0.431	0.725	21.3 ±1.1	10.1 ±0.4

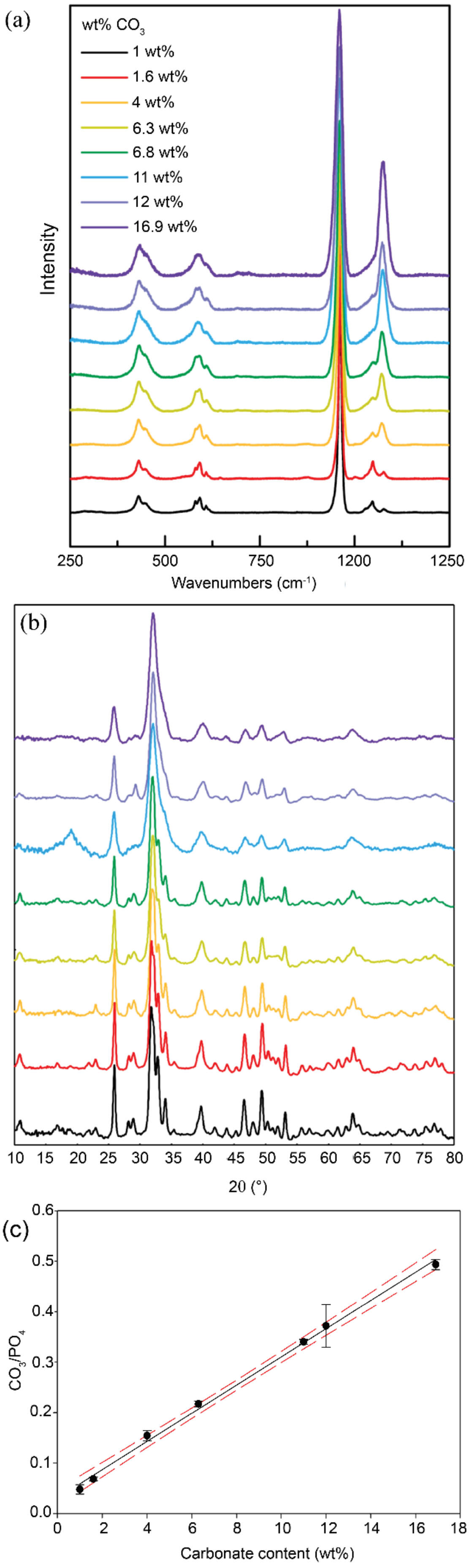

Figure S1 Raman spectra (a) and their associated powder XRD patterns (b) of synthetic carbonated apatite samples. (c) Carbonate content versus spectroscopic band CO_3/PO_4 ratio for determination of carbonate content from the mice specimens based on data from Raman spectroscopy. Spectra and diffraction patterns have been offset for clarity.

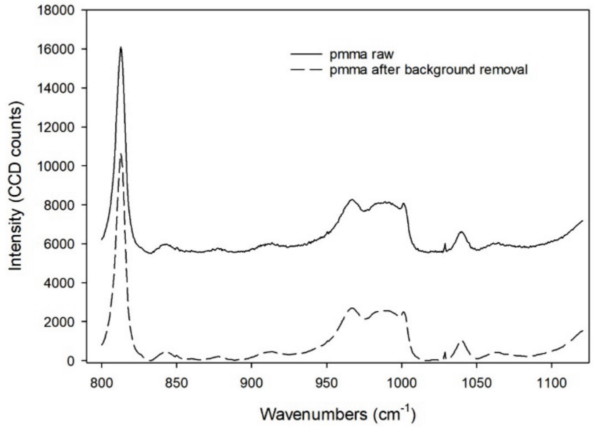

Figure S2 Raman spectra of the (poly)methyl methacrylate embedding material before and after background removal.

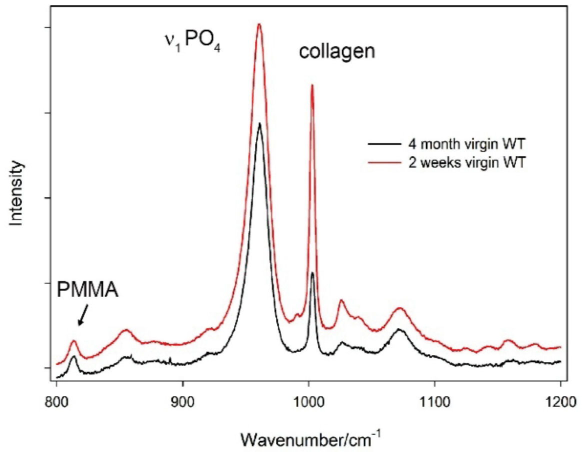

Figure S3 Raman spectra of virgin, normal mice showing the change in relative intensities between collagen related bands and those from the apatitic mineral phase related to specimen age at time of sacrifice. PMMA refers to the epoxy resin used to embed the sample. Spectra have been offset for clarity.

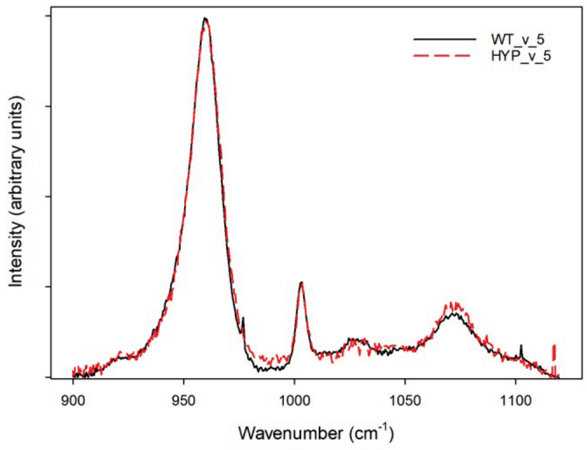

Figure S4 Comparison between virgin WT and HYP mice, age: five-months-old. Spectra have been normalised using the phosphate symmetrical stretching band at 960 cm^{-1} to aid comparisons.
